# Clinical Management of Medication-Assisted Treatment for Opioid Use Disorder Using a Mobile Health App Within a Primary Care Clinic: Quasi-Experimental Study

**DOI:** 10.2196/63526

**Published:** 2025-08-18

**Authors:** Allison D Rosen, Steven J Shoptaw, Li Li, Bengisu Tulu, Omar Nieto, Steven Jenkins, Mariah M Kalmin

**Affiliations:** 1 Department of Family Medicine, David Geffen School of Medicine, University of California Los Angeles, Suite 1800, 10880 Wilshire Blvd, Los Angeles, CA, 90024, United States, 1 3107946096; 2 Department of Psychiatry and Biobehavioral Sciences, David Geffen School of Medicine, University of California Los Angeles, Los Angeles, CA, United States; 3 The Business School, Worcester Polytechnic Institute, Worcester, MA, United States; 4 Q2i, Boston, MA, United States; 5 Department of Behavioral and Policy Sciences, RAND Corporation, Santa Monica, CA, United States

**Keywords:** mobile health, medication-assisted treatment, medication for opioid use disorder, mHealth, opioid use, primary care, mobile health app, software, assistance, United States, American, behavioral therapy, opioid use disorder

## Abstract

**Background:**

Medication-assisted treatment (MAT) is an effective strategy for treating opioid use disorder and reducing opioid-related overdose deaths, yet retention in treatment remains low. Mobile health (mHealth) platforms may be a useful tool for increasing long-term engagement in MAT programs, but evaluation studies of such platforms are limited.

**Objective:**

This study aimed to determine whether the use of the Opioid Addiction Recovery Support (OARS) software platform increased MAT engagement for patients with opioid use disorder.

**Methods:**

The Technology Improving Success of Medication-Assisted Treatment in Primary Care Study was a quasi-experimental study conducted at a primary care clinic in the United States between January 2021 and February 2022. OARS is a software platform and mobile app (Q2i, LLC) that includes a dashboard of real-time appointment attendance, urine toxicology (UTOX) results, and educational content as well as messaging and journaling features. All patients who were invited to use OARS and had available data across the study were included in the analysis. The primary outcomes were engagement in treatment, defined as no more than a 35-day gap in appointment attendance, and UTOX. Changes in treatment engagement between the treatment as usual (TAU) period and OARS intervention period were assessed using the effect size (Cohen *g*) and McNemar chi-square test of discordant pairs.

**Results:**

Among 205 patients invited to use OARS, 123 had available data and were thus included in the analysis. The median age was 37 (IQR 31‐42.5 ) years, 61% (75/123) identified as men, and 95.1% (117/123) identified as non-Hispanic White. There were no statistically significant differences in demographic characteristics for patients who used OARS on more than 1 day compared to patients who used OARS on 0 or 1 day, or patients who did versus did not have available data. Among all patients, 20% (25/123) were engaged in appointment attendance during TAU only compared to 27% (33/123) during OARS only (*g*=0.07; *P*=.36), and 13% (16/123) were engaged in UTOX during TAU only and 33% (41/123) during OARS only (*g*=0.21; *P≤*.01). Among a subsample of 52 patients who used OARS on more than 1 day, 17% (9/52) were engaged in appointment attendance during TAU only compared to 23% (12/52) during OARS only (*g*=0.07, *P*=.67), and 13% (7/52) were engaged in UTOX during TAU only and 35% (18/52) during OARS only (*g*=0.22, *P*=.05).

**Conclusions:**

Introduction of OARS in a primary care setting may be associated with a moderate change in MAT engagement as measured by UTOX, but not appointment attendance. While barriers to implementation and adoption, including difficulty fully integrating OARS with the clinic’s electronic health record, may have attenuated the potential effect of the intervention, this study provides evidence that mHealth interventions, such as OARS, are a promising addition to the MAT treatment landscape.

## Introduction

The United States is facing an unprecedented and growing opioid overdose epidemic, resulting in more than 80,000 deaths in 2022 [1]. Medication-assisted treatment (MAT), which involves the use of medications such as buprenorphine or methadone in combination with behavioral therapy and counseling, has proven effective for treating opioid use disorder (OUD) and reducing overdose deaths [2-4]. However, many patients do not remain engaged in MAT long-term due to a variety of psychological, social, and economic barriers [5-8].

Software and mobile health (mHealth) platforms offer a complementary service delivery modality for enhancing clinical management of OUD, increasing long-term engagement in MAT, and improving access to care [9-11]. A 2020 review did not find any evaluations of OUD-related mHealth apps [12]. Since then, multiple mobile apps have been identified as feasible and acceptable, including one that presents educational content and recent research (uMAT-r), one that facilitates patient-provider secure messaging (HOPE [Heal. Overcome. Persist. Endure.]), one that allows patients to track symptoms such as mood and cravings (KIOS), and an automated contingency management app that provides immediate monetary incentives for reaching treatment goals (PROCare Recovery) [13-17]. A 2024 scoping review of qualitative studies of OUD-related mHealth also reported high acceptability and perceived benefits by patients [18].

Research that builds on the feasibility and acceptability of these interventions by evaluating their effectiveness in improving OUD treatment outcomes remains limited, and findings are mixed. reSET-0, which uses neurobehavioral therapy techniques, was shown to reduce emergency and inpatient clinical encounters and increase treatment engagement, whereas the Addiction - Comprehensive Health Enhancement Support System (A-CHESS; CHESS Health) smartphone intervention was not associated with increased abstinence when added to medication treatment for OUD [19-21].

Given inconclusive findings from limited research on the effect of mHealth apps in improving OUD treatment outcomes, this analysis aims to contribute to the literature through a quasi-experimental pilot study evaluating the effect of the use of the Opioid Addiction Recovery Support (OARS) software on OUD treatment outcomes in a primary care setting. OARS is a software platform and mobile app (Q2i, LLC) that receives electronic health record (EHR) data to display a provider dashboard of real-time appointment attendance and urine toxicology (UTOX) results. Patients access the OARS mobile app on any smartphone where they can view their treatment records, message providers, interact with educational content including YouTube videos from the Substance Abuse and Mental Health Services Administration, and journal in a daily log. In addition to having access to a patient dashboard, providers can directly interact with patients via messaging and by viewing daily logs in order to inform treatment plans.

This study aimed to determine whether the use of the OARS software platform increased MAT engagement for patients receiving care at a primary care clinic in the United States.

## Methods

### Study Design

As part of a Small Business Technology Transfer Research Award, a partnership between a small business technology enterprise and an academic institution with research expertise, the Technology Improving Success of Medication-Assisted Treatment in Primary Care Study was conducted between January 2021 and February 2022 [22]. This quasi-experimental study observed patients receiving MAT at a primary care clinic in northeast Pennsylvania for 4 months (treatment as usual [TAU] period: January 1, 2021-April 30, 2021) before implementing the OARS platform clinic-wide for 10 months (OARS period: May 1, 2021-February 28, 2022).

No research staff were on site, and all interactions concerning the study occurred between patients and clinical staff (clinicians, nurses, case managers, etc). All patients receiving MAT at the clinic during the study period were eligible to participate, and the clinic’s 28 providers were asked to invite their MAT patients to sign up for OARS; patients were eligible to be included if they were an adult receiving care for OUD, had a smartphone, and agreed to download the OARS app and sign up for an account. The clinic extracted EHR data for all patients who initiated OARS accounts and delivered those data to Q2i during the TAU and OARS periods. Q2i then linked EHR data to OARS usage data, deidentified all data files, and provided the combined data to the research analytical team. All patients who signed up for OARS accounts and had EHR data provided by the clinic to Q2i throughout the study period were included in this analysis.

### Measures

The 2 primary outcomes of interest were consistent MAT engagement, as measured by (1) appointment attendance, defined as having no more than a 35-day gap between appointments, and (2) UTOX, defined as having no more than a 35-day gap between UTOX tests, with all UTOX tests being negative for opioids positive for buprenorphine, or both. A window of 35 days was chosen to allow for a 1-week grace period beyond the typical 28-day visit frequency.

### Statistical Analysis

Demographic characteristics including age, gender, and race or ethnicity were described using median and IQR values for continuous variables and counts and percentages for categorical variables. Due to implementation challenges that led to lower-than-expected rates of OARS usage, differences in demographic characteristics were compared for patients who used OARS on more than 1 day and patients who used OARS for 1 day or less, using Wilcoxon signed-rank tests for continuous variables and chi-square tests for categorical variables. A *P* value less than .05 was considered statistically significant.

The 2 outcomes of interest, engagement in MAT as measured by appointment attendance and engagement in MAT as measured by UTOX, were compared for the full sample as well as in a subsample of patients who used OARS regularly (ie, on more than 1 day). Each outcome was compared between the TAU and OARS periods using McNemar chi-square test of discordant pairs to account for paired data; a *P* value less than .03 was considered statistically significant as a Bonferroni adjustment for the 2 comparisons. Effect sizes were calculated for each outcome where *b* equals the number of patients engaged in MAT during the TAU period only and *c* equals the number of patients engaged in MAT during the OARS period only. Cohen *g* was calculated by computing *P*−.5, where *P* was defined as either *b/(b+c*) or *c/(b+c*), whichever was larger (*g*<0.15 was considered small, 0.15<*g*≤0.25 was considered moderate, and *g*>0.25 was considered large) [23]. All analyses were conducted in R (version 4.2.1; The R Foundation for Statistical Computing).

### Ethical Considerations

This study was approved by the University of California, Los Angeles Institutional Review Board with a waiver of informed consent (#20‐001516). All data were deidentified before being shared with the research team in order to maintain participant privacy. Participants were not compensated.

## Results

**Figure 1.**
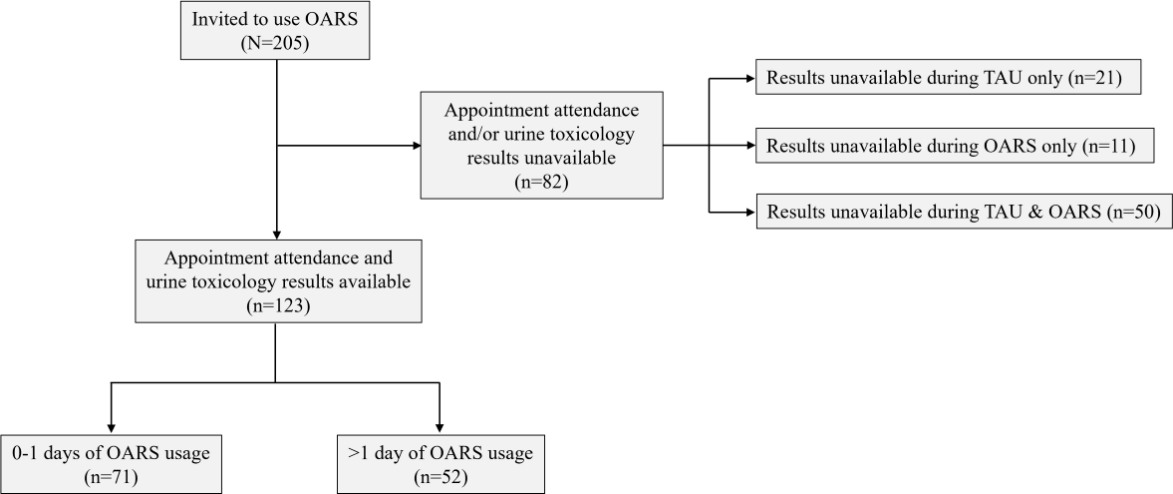
Flow diagram for the technology improving success of medication-assisted treatment in primary care study. OARS: Opioid Addiction Recovery Support; TAU: treatment as usual.

Among 205 patients invited to use OARS, the clinic provided appointment attendance and UTOX data during both the TAU and OARS periods for 123 patients (Figure 1). There were no differences in demographic characteristics or OARS usage for the 123 patients for whom the clinic provided data and the 82 for whom the clinic did not (Table 1).

Among the 123 patients analyzed, the median age was 37 (IQR 31‐42.5) years, 61% (75/123) identified as men, and 95.1% (117/123) identified as non-Hispanic White. Overall, 96 patients used OARS at least once: 99% (95/96) viewed test results at least once, 71.9% (69/96) viewed their treatment progress at least once, 67.7% (65/96) sent at least one message to their provider and averaged one message sent during the study, 66.7% (64/96) viewed educational content at least once, and 8.3% (8/96) created at least one daily log. There were no statistically significant differences in demographic characteristics for patients who used OARS on more than 1 day compared to patients who used OARS on 0 or 1 day (Table 2).

**Table 1.** Comparison of demographic characteristics and Opioid Addiction Recovery Support usage among patients for whom the clinic provided appointment and urine toxicology data (included in analysis) and those for whom the clinic did not (excluded from analysis).

Characteristics	Total (N=205)	Included in analysis (n=123)	Excluded from analysis (n=82)	*P* value
Age (years), median (IQR)	37 (31‐44)	37 (31‐42.5)	37 (32‐45.0)	.24
Gender, n (%)				
Male	130 (63.4)	75 (61)	55 (67.1)	.46
Race or ethnicity, n (%)				
Non-Hispanic White	193 (94.1)	117 (95.1)	76 (92.7)	.55
>1 day of OARS^a^ usage, n (%)	82 (40)	52 (42.3)	30 (36.6)	.50

a OARS: opioid addiction recovery support.

**Table 2.** Baseline characteristics of technology improving success of medication-assisted treatment in primary care study participants.

Characteristics	Total (N=123)	0‐1 days of OARS^a^ usage (n=71)	>1 days of OARS^a^ usage (n=52)	*P* value
Age (years), median (IQR)	37 (31‐42.5)	35 (29.5‐42.5)	37.5 (33.0‐42.3)	.23
Gender, n (%)				
Male	75 (61)	43 (60.6)	32 (61.5)	.91
Race or ethnicity, n (%)				
Non-Hispanic White	117 (95.1)	69 (95.8)	48 (92.3)	.37

aa OARS: opioid addiction recovery support

**Figure 2.**
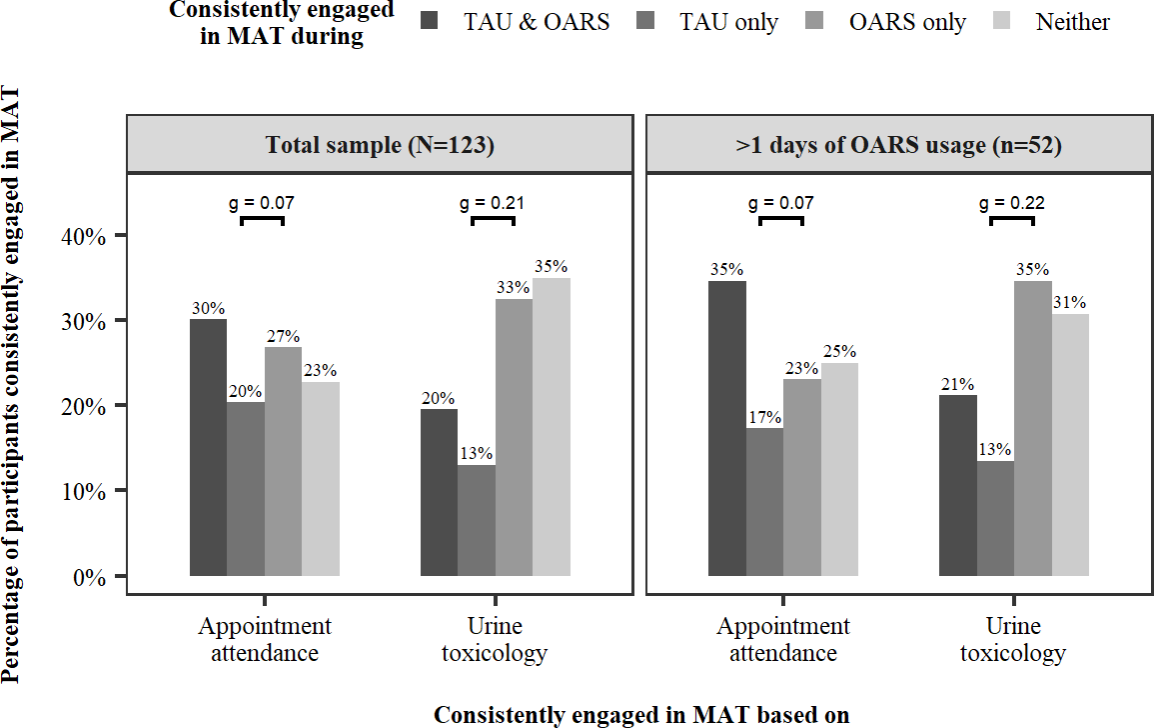
Medication-assisted treatment engagement during the study period. OARS: Opioid Addiction Recovery Support; MAT: medication-assisted treatment; TAU: treatment as usual.

Among the full sample of 123 patients, 20% (25/123) were engaged in appointment attendance during TAU only, compared to 27% (33/123) during OARS only, and 13% (16/123) were engaged in UTOX during TAU only and 33% (40/123) during OARS only; 7 participants (6%) tested positive for opioids over the course of the study, making the majority of UTOX engagement based on missed urine drug screens. There was no statistically significant difference in engagement in appointment attendance between the TAU and OARS periods, and the effect size was small (*g*=0.07, 95% CI −.06‐0.19; *P*=.36). There was greater UTOX engagement in the OARS period compared to the TAU period with a moderate effect size (*g*=0.21, 95% CI 0.09‐0.33; *P*<.01) (Figure 2).

Among 52 patients who used OARS on more than 1 day, 17% (9/52) were engaged in appointment attendance during TAU only compared to 23% (12/52) during OARS only, and 13% (7/52) were engaged in UTOX during TAU only and 35%(18/52) during OARS only. There was no statistically significant difference in engagement in appointment attendance between the TAU and OARS periods, and the effect size was small (*g*=0.07, 95% CI −0.15‐ to 0.29; *P*=.67). There was also no statistically significant difference in UTOX engagement between the TAU and OARS periods, and the effect size was moderate (*g*=0.22, 95% CI 0.02‐0.39; *P*=.05, ) (Figure 2).

## Discussion

### Principal Findings

This study found that the introduction of the OARS platform within a primary care clinic may be associated with increased MAT engagement as measured by UTOX, but not with increased MAT engagement as measured by appointment attendance. Moderate effect sizes were observed for changes in UTOX, and the difference was statistically significant in the full sample. The difference in the effect of OARS on engagement as measured by UTOX compared to appointment attendance warrants further exploration. This study did not collect additional information that could provide insight into this variation. Nonetheless, UTOX is a valid method to determine treatment engagement in the primary care setting and is therefore an appropriate outcome to assess [24].

Further investigation is needed to determine whether the discordance between the statistical tests in the full sample and the subsample of patients who used OARS on more than 1 day represents a pertinent difference or reflects a data issue, such as bias induced by data availability (Figure 1) or insufficient statistical power in the subsample. In addition, a 2021 evaluation of the reSET-O app (Pear Therapeutics) found increased engagement in service use (ie, case management, behavioral and mental health services) and decreased rates of drug testing, and a 2024 randomized clinical trial found that adding the A-CHESS smartphone intervention was not associated with increased abstinence from opioids when included as part of an MAT program [20,21].

While some of the seemingly opposite results from this study and previous studies may be explained by different focuses of the mHealth interventions as well as different treatment program structures and patient populations, the ways in which mHealth interventions for OUD may increase engagement in particular aspects of MAT programs must be studied further. In particular, because the current mHealth interventions for OUD vary widely in their methodology (ie, focus on patient-provider communication vs incentives vs neurobehavioral therapy), future studies should compare the acceptability, feasibility, and efficacy of existing platforms to determine how to create mHealth interventions for OUD that consistently improve treatment outcomes for patients.

The findings from this study may be explained by its limitations. This study experienced multiple implementation challenges, including unexpected inability to fully integrate OARS with the clinic’s EHR. This resulted in providers having to manually input information into OARS for patients instead of the data transferring automatically from the EHR, which in turn may have affected rates of usage by patients [22]. In addition, the majority of patients at this clinic were established maintenance patients who may have had low interest in and less need for a new recovery support tool. Furthermore, information was not available on patients who chose not to sign up for OARS, and it may be that more established patients who had high interest in care engagement were more likely to sign up. The sample size and limited background factors assessed in this study also made it unfeasible to account for confounding in analyses. Finally, the clinic was not able to provide EHR data for 82 participants. While measured demographic characteristics did not differ between these participants and those whose data were available, it is possible that unmeasured differences between the included and excluded groups could still be influencing the findings of this study.

### Conclusions

To our knowledge, this study is one of very few evaluations of mHealth platforms for the treatment of OUD in real-world settings that go beyond qualitative measures of acceptability and feasibility. While barriers to implementation and adoption may have impacted the ability to detect a strong effect of this intervention, this study provided the opportunity to introduce a first-of-its-kind app and provided valuable lessons for future implementation and platform development. The findings from this study reveal that OARS has the potential to be a promising contribution to the MAT treatment landscape, especially among patients who are new to MAT, and that mHealth should be further studied in the context of treatment for OUD. Future evaluations should consider ways to address implementation challenges as well as focus on patients who are in an earlier stage of treatment.

## Abbreviations

EHR: electronic health record
MAT: medication-assisted treatment
mHealth: mobile health
OARS: Opioid Addiction Recovery Support
OUD: opioid use disorder
TAU: treatment as usual
UTOX: urine toxicology

## References

[1] (2023). Drug overdose deaths: facts and figures. National Institute on Drug Abuse.

[2] Lagisetty P, Klasa K, Bush C, Heisler M, Chopra V, Bohnert A (2017). Primary care models for treating opioid use disorders: what actually works? A systematic review. PLoS ONE.

[3] Sordo L, Barrio G, Bravo MJ (2017). Mortality risk during and after opioid substitution treatment: systematic review and meta-analysis of cohort studies. BMJ.

[4] Herring AA, Rosen AD, Samuels EA (2024). Emergency department access to buprenorphine for opioid use disorder. JAMA Netw Open.

[5] Samples H, Williams AR, Olfson M, Crystal S (2018). Risk factors for discontinuation of buprenorphine treatment for opioid use disorders in a multi-state sample of Medicaid enrollees. J Subst Abuse Treat.

[6] Villamil VI, Underwood N, Cremer LJ, Rooks-Peck CR, Jiang X, Guy GP (2024). Barriers to retention in medications for opioid use disorder treatment in real-world practice. J Subst Use Addict Treat.

[7] Timko C, Schultz NR, Cucciare MA, Vittorio L, Garrison-Diehn C (2016). Retention in medication-assisted treatment for opiate dependence: a systematic review. J Addict Dis.

[8] Hall NY, Le L, Majmudar I, Mihalopoulos C (2021). Barriers to accessing opioid substitution treatment for opioid use disorder: a systematic review from the client perspective. Drug Alcohol Depend.

[9] Ganesh R, Rao R, Deb KS, Bhad R, Yadav D (2022). Digital capacity and interest in mHealth interventions among individuals on opioid agonist maintenance treatment: a cross-sectional community-based study. Indian J Psychol Med.

[10] Free C, Phillips G, Galli L (2013). The effectiveness of mobile-health technology-based health behaviour change or disease management interventions for health care consumers: a systematic review. PLoS Med.

[11] (2011). mHealth: new horizons for health through mobile technologies. World Health Organization African Region.

[12] Nuamah J, Mehta R, Sasangohar F (2020). Technologies for opioid use disorder management: mobile app search and scoping review. JMIR Mhealth Uhealth.

[13] Cavazos-Rehg PA, Krauss MJ, Costello SJ (2020). Delivering information about medication assisted treatment to individuals who misuse opioids through a mobile app: a pilot study. J Public Health (Oxf).

[14] Filiatreau LM, Szlyk H, Ramsey AT (2025). Sociodemographic differences in logins and engagement with the electronic health coach messaging feature of a mobile app to support opioid and stimulant use recovery: results from a 1-month observational study. JMIR Mhealth Uhealth.

[15] Waselewski ME, Flickinger TE, Canan C (2021). A mobile health app to support patients receiving medication-assisted treatment for opioid use disorder: development and feasibility study. JMIR Form Res.

[16] King VL, Siegel G, Priesmeyer HR, Siegel LH, Potter JS (2024). Development and evaluation of a digital app for patient self-management of opioid use disorder: usability, acceptability, and utility study. JMIR Form Res.

[17] Proctor SL, Rigg KK, Tien AY (2022). Acceptability and usability of a reward-based mobile app for opioid treatment settings: mixed methods pilot study. JMIR Form Res.

[18] Lyzwinski LN, Elgendi M, Menon C (2024). Users’ acceptability and perceived efficacy of mHealth for opioid use disorder: scoping review. JMIR Mhealth Uhealth.

[19] Christensen DR, Landes RD, Jackson L (2014). Adding an internet-delivered treatment to an efficacious treatment package for opioid dependence. J Consult Clin Psychol.

[20] Velez FF, Colman S, Kauffman L, Ruetsch C, Anastassopoulos K (2021). Real-world reduction in healthcare resource utilization following treatment of opioid use disorder with reSET-O, a novel prescription digital therapeutic. Expert Rev Pharmacoecon Outcomes Res.

[21] Gustafson DH, Landucci G, Vjorn OJ (2024). Effects of bundling medication for opioid use disorder with an mHealth intervention targeting addiction: a randomized clinical trial. Am J Psychiatry.

[22] Nieto O, Rosen AD, Kalmin MM (2025). Adoption of a digital health tool for opioid use disorder treatment in primary care: facilitators and challenges (preprint). Journal of Medical Internet Research.

[23] Cohen J (1988). Statistical Power Analysis for the Behavioral Sciences.

[24] Biondi BE, Zheng X, Frank CA, Petrakis I, Springer SA (2020). A literature review examining primary outcomes of medication treatment studies for opioid use disorder: what outcome should be used to measure opioid treatment success?. Am J Addict.

